# Death after an accidental fall of a 101 year old hospitalized patient. Medico-legal implication of falling in geriatrics

**DOI:** 10.1186/1471-2482-12-S1-S34

**Published:** 2012-11-15

**Authors:** Claudio Terranova, Fabrizio Cardin, Bruno Martella, Bruno Amato, Carmelo Militello

**Affiliations:** 1 Department of Molecular Medicine, University of Padua, Italy; 2 Department of Surgical and Gastroenterological Sciences, University of Padua, Italy; 3 University of Naples Federico II - Department of General Surgery, Italy

## Abstract

**Background:**

The case presented by the authors gives the opportunity to discuss the medico-legal issues related to lack of prevention of falls in elderly hospitalized patients.

**Case presentation:**

A 101 year old Caucasian female was admitted to a surgery division for evaluation of abdominal pain of uncertain origin. During hospitalization, after bilateral bed rails were raised, she fell and reported a femoral fracture. Before surgical treatment of the fracture, scheduled for the day after injury, the patient reported a slight reduction in hemoglobin. She received blood transfusion but her general condition suddenly worsened; heart failure was observed and pulseless electrical activity was documented. The patient died 1 day after the fall. Patient relatives requested a judicial evaluation of the case.

The case was studied with a methodological approach based on the following steps: 1) examination of clinical records; 2) autopsy; 3) evaluation of clinicians’ behavior, in the light of necroscopic findings and a review of the literature.

**Conclusions:**

The case shows that an accurate evaluation of clinical and environmental risk factors should be always performed at the moment of admission also in surgery divisions. A multidisciplinary approach is always recommended also with the involvement of the family members. In some cases, as in this one a fall of the patient is expectable but not always avoidable. Physical restraint use should be avoided when not necessary and used only if there are no practical alternatives.

## Background

A fall [1], an event which results in a person coming to rest inadvertently on the ground or floor or other lower level, is the most commonly reported adverse event in hospitals and nursing homes. It mainly affects the elderly and cause of disability, morbidity and mortality [2]. The deterioration of quality of life following a fall is related to the onset of fear of new falls [3], loss of confidence, anxiety, depression, and reduced autonomy. Mental and physical damage is also associated with an increased length of hospitalization, additional diagnostic and therapeutic interventions, and further admissions after discharge, with an increase in health and social costs.

It is estimated that about 14% of falls during hospitalization may be classified as accidental, due to environmental factors (e.g. slipping on the wet floor), 8% as physiological and unpredictable, due to sudden mutation of patient physical condition (e.g., sudden balance disorder), and 78% of falls as physiological but predictable due to identifiable risk factors (e.g. patient disoriented, with walking difficulties) [4].

Risk patient identification is the first preventive step that should be considered. The systematic assessment of clinical and environmental risk factors must be implemented at admission and during hospitalization especially in case of modification of patient condition.

The assessment of the risk of falling may be done through a systemic evaluation on individual and environmental risk factors [5-7] integrated by scales and tests for the detection of the risk of falling. Older age, multiple diseases, history of falls, gait disorders and/or weakness in the lower limbs, incontinence or frequent urination, cognitive impairment (disorientation, restlessness, confusion and impaired judgment), psychotropic drugs, impaired vision, need help in postural changes, are individual risk factors of falling during hospitalization. Prolonged hospitalizations, some environments such as the bathroom or the zone near the bed and some time slots with the presence of few staff member are environmental risk factors of falling [8].

Once you have identified risk factors it is essential that providers, patients and family/caregivers acquire the awareness of the risk of falling and work together in an integrated and consistent, careful application of multifactorial strategies.

Interventions after risk assessment may reduce but not eliminate the risk of falling. A fall of a patient during hospitalization may have not only clinical consequences for the patient but also judicial consequences for the clinician.

The case presented by the authors gives the opportunity to discuss the medico-legal issues related to falls in elderly hospitalized patients. A systematic review on the thematic of falls is beyond the purpose of this paper and we refer to the numerous national [9] and international guidelines [5,10] for a comprehensive discussion of this topic.

## Methods

The case was studied with a methodological approach based on the following steps: 1) examination of clinical records; 2) autopsy; 3) evaluation of physicians’ behavior, in the light of necroscopic findings and a review of the literature.

### Clinical documentation

A 101 years old Caucasian female was admitted to a surgery division for evaluation of abdominal pain of uncertain origin [11]. She was affected by hypertension, heart failure, macrocytic anemia, initial cognitive impairment associated to transient confusional states, urinary incontinence and previous old femoral fracture of the right limb. Walking was possible only through the help of a family member or nurse.

The initial cognitive impairment associated to confusional states, the walking difficulties, the lack of personnel or family members capable of monitoring the patient during the night induced clinicians to use physical restraint and to ask nurses a more frequent evaluation of the patient. Bilateral bed rails were raised. During the morning of the next day of hospitalization, the nurse heard a noise coming from the room of the patient finding her on the floor. Immediate examination and instrumental evaluation allowed diagnosing a left femoral fracture and a slight concussion. TC scan excluded skull fractures or intracranial hemorrhages. A surgical treatment intervention was scheduled for the next day but during the afternoon the clinical condition worsened. Laboratory analysis revealed slight low levels of hemoglobin (92 g/l (reference values of 123-153 g/L) and a worsening of neurological condition. The physician decided to transfuse the patient with two units of packed red blood cells. During the night a deterioration of the overall clinical picture was observed with dyspnea and bradycardia not responding to the treatments. Pulseless electrical activity (PEA) was documented; cardiopulmonary resuscitation measures were performed with no result. She was declared dead in the morning of the day after the fall.

### Necroscopic data

External examination showed at palpation signs of fracture of thorax and on the left lower hip. Section revealed a picture of cerebral oedema, bilateral rib fractures due to resuscitation, myocardial fibrotic areas, congestion and emphysema of the lungs, polyvisceral congestion, polydistrectual atherosclerosis.

Histological examination confirmed the macroscopic findings, demonstrated the presence of interstitial myocardial fibrosis in absence of signs of recent myocardial infarct; significant areas of emphysema and congestion of the lung, areas of glomerulosclerosis were put in evidence.

### Cause of death

The cause of death was identified in cardio-circulatory arrest in cardiogenic shock, pulseless electrical activity in recent left femoral fracture, ischemic-hypertensive chronic heart disease, lung emphysema, chronic macrocytic anemia and previous old right femoral fracture.

## Discussion and conclusions

In Western countries, the aging of the population leads to an increase in the number of elderly patients admitted to hospitals or nursing homes. The growing number of medico-legal cases concerning professional liability [12] may represent a cause of increase of litigation also in cases involving elderly patients. Older patients have more co-morbid conditions but an accurate selection of the patients before operations may prevent or reduce complications [13] after surgery and consequently the possibility of litigations.

The methodological approach of evaluation of professional liability cases is based on the definition of damage to the patient (diagnosis of a disease or identification of the cause of death), on the analysis of the conduct of clinicians and on verification of causal relation between patient damage and the possible error of clinicians [14,15].

The identification of the cause of death requires a necroscopic examination [16]. Autoptic studies conducted on elderly are uncommon [17] and those with any legal purposes are even rarer [18] and related to cases of murder and suicide and rarely to professional liability hypothesis.

In our case the claim of malpractice was promoted by the relatives of the deceased. Prosecution was based on the hypothesis of a lack of fall prevention and on an incorrect use of physical restraint.

According to the methodological approach clinical documentation analysis and autopsy were performed. The cause of death was linked with no doubt to the fall that triggered, acting on baseline patient health condition, a lethal pathophysiological evolution. Patient general clinical conditions were in precarious balance - she was very old and suffering from multiple diseases – and bleeding from fracture was considered a sufficient factor to alter patient precarious equilibrium with heart failure which hesitated rapidly in the death of the patient.

With reference to the malpractice claim, the conduct of clinicians was considered correct. Initial cognitive impairment associated to transient confused states, urinary incontinence and walking difficulties were considered by clinicians as risk factors for fall. The awareness of risk of falling finds demonstration in the use of physical restraints. The clinicians considered properly the risk factors providing guidance to the nursing staff for the proper management of postural changes of the patient and of the aid to be provided during postural changes. Greater oversight of patients with more frequent rounds of inspection were established. The use of side rails during the night was considered a necessary measure to limit the ability of movement of the patient and nocturnal wanderings. This choice was widely discussed during the trial. According to prosecution, physical restraints such as bilateral bed rails, belts, and fixed tables in a chair should be always considered inadequate or wrong. Physical restraints are quite frequently used in geriatric division in Italy despite some evidences for their lack of effectiveness and safety [19-21]. In US a recent survey reported physical restraint rates of more than 20% in nursing homes [22]. The authors agree with the opinion of some authors [23,24] that physical restraint could be considered acceptable only when specific benefits are envisioned and there are no practical alternatives to physical restraint. If physical restraint is applied, clinicians should adopt all additional measures necessary to respect the dignity of the patient and to avoid complications related to the treatment [22]. Continuous monitoring, as in this case and as frequently as possible, should be performed. Interruption of physical restraints or re-evaluation of the justification for physical restraint should be performed at regular intervals [22,24].

The choice of using physical restraint was considered a forced choice in our case due to the high risk of fall and the lack of sufficient personnel to monitor the patient during the day.

The case shows that an accurate evaluation of clinical and environmental risk factors should be always performed at the moment of admission of the patient also in a surgery division. A multidisciplinary and multicomponent intervention is always recommended also with the involvement of the family members. In some cases a fall of the patient is expectable but not always avoidable. In certain situations, the lack of adequate number of personnel, or of family members who may look after the patient, does not exclude the risk of fall. Physical restraint use should be avoided when not necessary and used only if there are no practical alternatives.

## Authors’ contributions

CT performed the autopsy, contributed to the analysis and interpretation of the data, to the discussion of medico-legal issues and to the writing of the paper. FC contributed to the literature review, to the drafting and reviewing of the paper. BM, BA and CM gave their contribution to the analysis of the conduct of clinicians and contributed to the writing of the paper. All the authors read and approved the final manuscript.

## Competing interests

The authors declare that they have no competing interests.
